# A qPCR identification scheme to differentiate actinomycetoma from eumycetoma

**DOI:** 10.1007/s10096-026-05572-8

**Published:** 2026-06-26

**Authors:** Sahar Bakhiet, Emmanuel Siddig, Carole Pab Minlekib, Rihab Mohammed, Willemien Zandijk, Andrew K. Watson, Mickey Konings, Ahmed Fahal, Maguette Faye, Fatehmeh Mazraati Tajabadi, Jeffrey Errington, Doudou Sow, Wendy W. J. van de Sande

**Affiliations:** 1 Department of Medical Microbiology and Infectious Diseases, ErasmusMC, Dr. Molewaterplein 40, 3015 GD Rotterdam, Netherlands; 2 Mycetoma Research Centre, University of Khartoum, Khartoum, Sudan; 3 The Institute of Endemic Diseases, University of Khartoum, Khartoum, Sudan; 4 Faculty of Medical Laboratory Sciences, University of Khartoum, Khartoum, Sudan; 5 Centre de Recherche sur les maladies Tropicales Négligées, UFR Sciences de la Santé, Université Gaston Berger, Saint-Louis, Senegal; 6 Department of Medical Microbiology and Parasitology, Faculty of Medical Laboratory Sciences, University of Dongola, Northern, Sudan; 7 Centre for Bacterial Cell Biology, Biosciences Institute, Newcastle University, Newcastle upon Tyne, UK; 8 Faculty of Medicine and Health, University of Sydney, Sydney, NSW 2015 Australia

**Keywords:** Mycetoma, Eumycetoma, Actinomycetoma, qPCR, Target product profile, Diagnostics, Epidemiology

## Abstract

**Background:**

Mycetoma is a Neglected Tropical Disease of the skin. It is characterized by large tumorous lesions in the subcutaneous tissue which hinder the patients in their daily activities. The disease can be caused by either bacteria (actinomycetoma) or fungi (eumycetoma), and treatment depends on the entity. Therefore, in 2022, a Target Product Profile was published by the World Health Organisation in which it was stated that there is an urgent need to develop a diagnostic tool that can differentiate the two reliably within a day.

**Objectives:**

To develop a qPCR that can not only differentiate actinomycetoma from eumycetoma but can also identify the actinomycetoma causative agents to the genus level.

**Methods:**

We developed singleplex, quadruplex and triplex qPCRs that can be used, depending on the endemic region and validated this against a large strain collection and grains.

**Results:**

The limit of detection (LOD) of each of the probes ranged from 0.000002 ng for the fungal probe to 0.002 ng for the *Nocardia* genus probe in a singleplex reaction. Higher LODs were obtained in the multiplexes. The sensitivity and specificity were both 100% for most combinations, although some cross-reactivity with the *Actinomadura* and *Streptomyces* genus probes was noted, mainly with non-mycetoma causative agents.

**Conclusion:**

The developed qPCR meets the requirements set in the Target Product Profile from the World Health Organisation.

**Importance:**

In this study a qPCR is developed which can differentiate actinomycetoma from eumycetoma and identify actinomycetoma causative agents to the genus level. This qPCR adheres to the minimal requirements set in the Target Product Profile for a mycetoma diagnostic test by the World Health Organisation. When the qPCR is used on mycetoma grain it reduces the time to identification to only a few hours. With hand-held qPCRs this can also be performed in the field.

**Supplementary Information:**

The online version contains supplementary material available at https://doi.org/10.1007/s10096-026-05572-8.

## Introduction

Mycetoma is a Neglected Tropical Disease of the skin [1]. It is characterized by large tumorous lesions in the subcutaneous tissue, which hinder the patients in their daily activities [1]. Mycetoma can be caused by 90 different agents, either bacteria (actinomycetoma) or fungi (eumycetoma) [2]. Characteristic of the mycetoma causative agents is that they form granules in the tissue called grains [2]. Accurate and rapid differentiation between actinomycetoma and eumycetoma is crucial, as their treatments differ considerably. Actinomycetoma is treated in five-week antibiotic cycles and 90% of patients reach complete cure within three 5-week cycles [3, 4]. Eumycetoma is managed with a combination of antifungal treatment and surgery [5]. Under the conditions of a clinical trial this resulted in cure for 80% of patients [5]. In case series this resulted in a 56% corrected cure rate [6].

Due to the critical importance of prompt and accurate diagnosis to initiate the appropriate treatment, the World Health Organization (WHO) has established a Target Product Profile (TPP) emphasizing the need for an accessible in vitro point-of-care capable of identifying mycetoma causative agents to the species level [7]. Ideally, this test would operate under zero infrastructure conditions and identifies all causative agents to the species level. With 90 different causative agents, not an easy task to develop. A more achievable goal would be to develop a laboratory-based test which could differentiate actinomycetoma from eumycetoma within a day, and which is performed in a peripheral health facility or national diagnostic testing laboratory would. Such a test would meet the minimal requirements as stipulated in this TPP [7].

Currently, diagnosis in endemic countries relies on clinical examination, histopathology, and culture [8], which results in an average time to identification of 8.5 days for histopathology and of 21.2 days for culture [9]. This exceeds the time set in the minimal requirements in the TPP. Furthermore, when the performance of these assays was validated in a cohort of 222 eumycetoma patients, it appeared that for histopathology a sensitivity of 93.8% and specificity of 42.8% was obtained and for culture a sensitivity of 84.5% and specificity of 85.7% [9]. The sensitivity of histopathology was in general lower for actinomycetoma patients caused by *Streptomyces somaliensis* (*n* = 142 cases; 93.7% sensitivity and 98.9% specificity), *Actinomadura madurae* (*n* = 71 cases; 74.4% sensitivity and 99.5% specificity) or *Actinomadura pelletieri* (*n* = 16 cases; 75.0% sensitivity and 100% specificity) [10]. For eumycetoma, molecular diagnostic assays such as PCR and qPCR were developed [11–14], which can identify the most common fungal causative agent *Madurella mycetomatis* in 2.5 h [9]. Using these PCRs a sensitivity of 98.9% and specificity of 100% was obtained in the same eumycetoma cohort of 222 patients [9]. For actinomycetoma, no species-specific PCRs have been developed for most of the causative agents and molecular identification to the species level could only be performed for *Nocardia* by using a genus specific PCR [15] or by using either a barcode PCR followed by restriction fragment length polymorphism analyses or sequencing or by whole genome sequencing directly from patient material [16–19]. These techniques are often not available in endemic countries and still require more than 1 day for identification.

During the SARS-COVID2 pandemic, many endemic countries obtained qPCR machines [20]. After the pandemic, these machines remained operational and could be used for other diagnostic purposes. We therefore aimed to develop a qPCR which could differentiate eumycetoma from actinomycetoma. Since bacterial co-infections are commonly found in mycetoma lesions, a pan-bacterial probe could not be used [21]. Since more than 95% of the actinomycetoma causative agents belong either to the bacterial general *Actinomadura*,* Nocardia* or *Streptomyces*, we therefore aimed to develop a multiplex qPCR which would differentiate eumycetoma from actinomycetoma caused by *Actinomadura* species, *Nocardia* species and *Streptomyces* species [2].

## Materials and methods

### Bacterial and fungal strains

For this study, we used a collection of 91 bacterial and 63 fungal isolates of mycetoma causative agents (Table 1, Supplementary Table 1) and *Staphylococcus aureus* and *Mycobacterium tuberculosis* as negative controls. The isolates were obtained from culture collections such as the germen collection of micro-organsims (DSMZ) or the Westerdijk fungal collection (CBS) or from patients seen at the Mycetoma Research Centre in Sudan or the University of Saint-Berger in Senegal. The details about the origin of each isolate are provided in Supplementary Table 1. Additionally, 10 grain samples (5 bacterial grains and 5 fungal grains) were obtained from 10 mycetoma patients from University Saint-Berger in Senegal. This study was performed in line with the principles of the Declaration of Helsinki. Approval was granted by the National Ethical Committee of Senegal (Comité National dÉthique pour la Recherce en Santé; CNERS) (No 0000063/MSAS/CNERS/SP) for the Senegalese isolates and from the Soba University Hospital Ethical committee (Khartoum, Sudan) for the Sudanese isolates. Informed consent was obtained from all individual participants included in the study.

**Table 1:** Isolates used in this study

Species	Bacterial/Fungal	Known mycetoma causative agent#	Number of isolates
*Actinocorallia weidayensis*	Bacterial	No*	1
*Actinomadura cremea*	Bacterial	No	1
*Actinomadura geliboluensis*	Bacterial	Yes	2
*Actinomadura hallensis*	Bacterial	No	1
*Actinomadura latina*	Bacterial	Yes	1
*Actinomadura madurae*	Bacterial	Yes	19
*Actinomadura meyerae*	Bacterial	No	2
*Actinomadura pelletieri*	Bacterial	Yes	13
*Actinomadura welshii*	Bacterial	Yes	2
*Nocardia asteroides*	Bacterial	Yes	1
*Nocardia brasiliensis*	Bacterial	Yes	9
*Nocardia farcinica*	Bacterial	Yes	1
*Nocardia mexicana*	Bacterial	Yes	1
*Nocardia vulneris*	Bacterial	Yes	2
*Sphaerosporangium sp.*	Bacterial	No	1
*Streptomyces albidoflavus*	Bacterial	No*	2
*Streptomyces albogriseuolus*	Bacterial	No*	1
*Streptomyces chrestomyceticus*	Bacterial	No	1
*Streptomyces griseus*	Bacterial	Yes	1
*Streptomyces kordofanensis*	Bacterial	No*	1
*Streptomyces radiopugnans*	Bacterial	No*	1
*Streptomyces somaliensis*	Bacterial	Yes	7
*Streptomyces sudanensis*	Bacterial	Yes	19
*Streptomyces werraensis*	Bacterial	No*	1
*Exophiala oligosperma*	Fungal	No*	1
*Falciformispora senegalensis*	Fungal	Yes	6
*Falciformispora tompkinsii*	Fungal	Yes	2
*Madurella fahalii*	Fungal	Yes	3
*Madurella mycetomatis*	Fungal	Yes	35
*Madurella pseudomycetomatis*	Fungal	Yes	7
*Madurella tropicana*	Fungal	Yes	2
*Medicopsis romeroi*	Fungal	Yes	1
*Nigrograna mackinnonii*	Fungal	Yes	1
*Scedosporium boydii*	Fungal	Yes	1
*Trematosphaeria grisea*	Fungal	Yes	4

#A species was considered a known causative agent of mycetoma if it was published in at least a case report as being a mycetoma causative agent

*Although these species were not known as mycetoma causative agents, the isolates used in this study were isolated from mycetoma patients

From each of these patients informed consent was obtained. Bacterial isolates were maintained on BBL™ Trypticase™ Soy Agar with 5% Sheep Blood (Becton Dickinson, USA) at 37 ᵒC. Fungal isolates were maintained on Sabouraud Dextrose Agar (Becton Dickinson, USA) at 37ᵒC or 21ᵒC depending on the species.

### DNA isolation

DNA was isolated from a 0.25cm^2^ fungal colony, three bacterial colonies or a single fungal grain using the Fungal/Bacterial DNA MicroPrep kit (Zymo Research, USA). The DNA concentration was determined using the nanodrop and the Quant-iT™ PicoGreen™ dsDNA Assay Kit (Invitrogen, USA).

### Identification of genus-specific target region, design and validation of the qPCR

For fungi the internally transcribed spacer (ITS) region serves as a widely used barcoding marker, capable of amplifying a broad range of fungal species. For actinomycetes either 16 S rRNA or heat shock protein 65 (*HSP65*) are used as a barcoding genes [22]. To design a genus-specific qPCR, the highly conserved HSP65 sequences of 69 different *Actinomadura*, *Nocardia*, *Mycobacteria* and *Streptomyces* species were retrieved from GenBank (Supplementary Table 2). The sequences were aligned in MEGA-X. Highly conserved regions within the *Actinomadura* genus, *Nocardia* genus and *Streptomyces* genus were used to design the primer regions. For the fungal species we compared the ITS regions and selected a highly conserved region.

The representative *M. mycetomatis*,* A. madurae*,* N. brasiliensis* and *S. somaliensis* sequences were loaded into Primer3 (version 0.4.0; https://bioinfo.ut.ee/primer3-0.4.0/). Optimum Tm was set at 60 °C (± 3 °C) for the primers and at 70 °C (± 3 °C) for the probes. A multiple primer dimer analysis was performed using the Multiple Prime Analyzer tool (https://www.thermofisher.com/nl/en/home/brands/thermo-scientific/molecular-biology/molecular-biology-learning-center/molecular-biology-resource-library/thermo-scientific-web-tools/multiple-primer-analyzer.html). The variable region between the primers was used to design genus-specific probes. Primers and probes are listed in Table 2.

**Table 2:** Selected primers and probes

Target	Name	Sequence	Concentration (µM)	Quadruplex (µl)	African triplex (µl)	Latin-American triplex (µl)	Actinomycetoma triplex (µl)
Fungi	Panfungal_ITS_Fw1	5’-TCTGGCATCGATGAAGAA − 3’	50	0.25	0.25	0.25	
Fungi	Panfungal_ITS_RV1	5’-TGACGCTCGAACAGGCAT − 3’	50	0.25	0.25	0.25	
Fungi	EumycPanfungal_ProbeITS	5’ HEX -TTTGAACGCACATTGCGCC -BHQ^®^1–3’	20	0.1	0.1	0.1	
*Actinomadura* and *Streptomyces* genus	Act_Hsp65_fw	5’-GTGGAGACCAAGGAGCAGAT-3’	50	0.25	0.25	0.25	0.25
*Actinomadura* and *Streptomyces* genus	Act_Hsp65_rv	5’-CTTCCTTGCCGACCTTGTC-3’	50	0.25	0.25	0.25	0.25
*Actinomadura* genus	Actinomadura_Hsp65_probe	5’-TEXAS RED^®^-ACCCGCAGATCGGCGAGATGAT-BHQ^®^2–3’	20	0.1	0.1	0.1	0.1
*Streptomyces* genus	Streptomyces_Hsp65_probe	5’-6-FAM -CCGCCTCCATCTCCGCC- ECLIPSE^®^ DARK QUENCHER-3’	20	0.1	0.1		0.1
*Nocardia genus*	Nocardia_hsp65_fw	5’-GATCGATACCAAGGAGCAGAT-3’	50	0.25		0.25	0.25
*Nocardia genus*	Nocardia_hsp65_rv	5’-CTTCCTTGCCGACCTTGTC-3’	50	0.25		0.25	0.25
*Nocardia genus*	Nocardia_Hsp65_probe	5’-CY 5^®^-CGTCCATCGGTGAGCTCATCGC-BHQ^®^3–3’	20	0.1		0.1	0.1
DNA free water				6.1	6.7	6.2	6.7
LightCycler 480 Probes Master Mix				10	10	10	10

Primers were validated against a small subset of DNA samples with classical PCR. For this a master mix was prepared consisting of 0.25µL of forward primer (50µM), 0.25µL of reverse primer (50 µM), 10 µL of Roche FastStart™ PCR Master and 7.5 µl sterile deionized water. 18µL of the mix was transferred to a PCR tube (Bio-Rad, Lunteren, The Netherlands) containing 2µL of 2.5ng/µl bacterial or fungal DNA. The PCR was performed using a conventional PCR machine (Bio-Rad, Lunteren, The Netherlands), with a hot start at 94 °C for 5 min, followed by 40 cycles of 94 °C, melting for 30s, 60 °C annealing for 30s, and 72 °C elongation for 1 min. The amplicons were then visualized by gel electrophoresis on a 2.5% agarose (Sphaero Q, Gorinchem, The Netherlands ) stained with SYBR safe (Invitrogen, Eindhoven, The Netherlands). A 100 bp DNA ladder (Invitrogen, Eindhoven, The Netherlands) was used as a size reference.

### Validation of singleplex qPCR

To assess the probe, a master mix was prepared consisting of 0.25 µL forward primer (50 µM), 0.25 µl reverse primer (50 µM), 0.1 µL probe (20 µM), 10 µL of LightCycler 480 Probes Master Mix and 7.4 µl DNA-free water. Then 18 µL of master mix was transferred to a 96-wells plate (LightCycler^®^ 480 multiwell, Roche, Basel, Switzerland) and mixed with 2 µL of DNA sample and run on the LightCycler^®^ 480 (Roche, Basel, Switzerland) with an amplification program consisting of a 5 min preheating step at 95 °C, followed by 40 cycles consisting each of a 5s DNA denaturation step at 95 °C, and a 30s annealing and extension step at 60 °C, and ending with a 1 min cooling step at 40 °C. The fluorescence output was measured at 465–510 nm, 533–580 nm, 595–615 nm and 618–660 nm and analyzed using the Abs Quant/Fit Points in the LightCycler 480SW 1.5.1 program (Roche, Basel), using 35 cycles as cut-off.

### Validation of the multiplex qPCR

The multiplex qPCR was validated in four distinct steps. First, the probes were screened against a small subset of DNA samples in singleplex and multiplex to confirm the intended functionality of the probes. Second, the multiplex qPCR was screened against a larger DNA collection listed in Table 1. Third, the limit of detection (LOD) was determined based on a 10-fold dilution series ranging from 0.00001 ng to 10 ng of DNA isolated from *A. madurae* DSM43067, *A. pelletieri* DSM43383, *S. somaliensis* DSM40738 and *S. sudanensis* DSM41923. The LOD was determined for all three probes in triplicate, in singleplex, and multiplex. The data was analyzed as described above, and the Cq values of each sample were plotted against the respective DNA concentration and visualized for each of the respective probes. Finally the multiplex was validated against DNA isolated from 10 grain samples (5 bacterial grains and 5 fungal grains). The amount of each primer and probe used in the different multiplex qPCR set-ups are listed in Table 2. For each multiplex an amplification program consisting of a 5 min preheating step at 95 °C, followed by 40 cycles consisting each of a 5s DNA denaturation step at 95 °C, and a 30s annealing and extension step at 60 °C, and ending with a 1 min cooling step at 40 °C was used. The fluorescence output was measured at 465–510 nm, 533–580 nm, 595–615 nm and 618–660 nm and analyzed using the Abs Quant/Fit Points in the LightCycler 480SW 1.5.1 program (Roche, Basel), using 35 cycles as cut-off.

### Statistical methods

To determine the sensitivity, specificity, positive predictive value and negative predictive value of the singleplex and multiplex qPCRs, the number of true-positive (TP), false-positive (FP), true-negative (TN) and false-negative (FN) test results were analysed with the chi-squared test (Graphpad).

## Results

### The primers developed on ITS and HSP65 could be used to amplify the fungal and bacterial causative agents of mycetoma, respectively

**Fig. 1:**
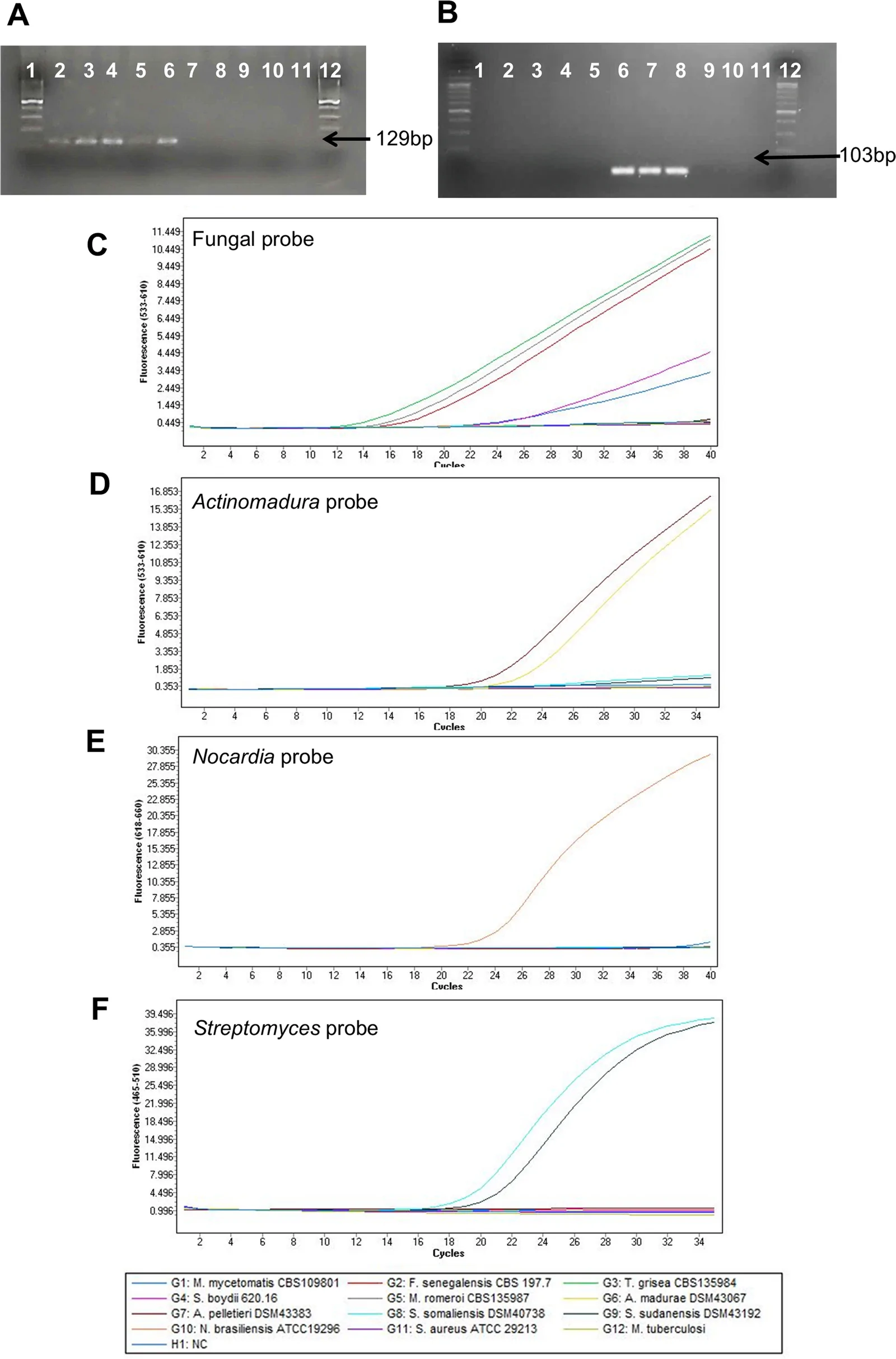
qPCRs to differentiate eumycetoma and actinomycetoma causative agents.** A**: Gel electrophoresis demonstrating PCR products generated with the designed ITS forward and reverse primers. **B**: Gel electrophoresis demonstrating PCR products generated with the designed HSP65 forward and reverse primers. The order of the amplification products in panels **A **and** B** is as follows: in lane 1 and 12, a 100 bp ladder is shown. In lanes 2 to 10, the amplification products of the various mycetoma causative agents are shown. These are amplification products for *M. mycetomatis* CBS109801 (lane 2), *F. senegalensis* CBS 197.79 (lane 3), *T. grisea* CBS135984 (lane 4), *S. boydii* CBS620.16 (lane 5), *M. romeroi* CBS135987 (lane 6), *A. madurae* DSM43067 (lane 7), *S. somaliensis* DSM40738 (lane 8), *N. brasiliensis* ATCC19296 (lane 9), *S. aureus* ATCC29213 (lane 10). The negative control is depicted in lane 11. **C**: qPCR using ITS primers and the ITS probe. The fluorescent signal was plotted against cycles. Fluorescent signal measured at 533–580 nm corresponding to the HEX fluorophore of the ITS probe. **D**: qPCR using HSP65 primers and the *Actinomadura* probe. Fluorescent signal measured at 595–615 nm corresponding to the TexasRed fluorophore of the *Actinomadura* probe. **E**: qPCR using HSP65 primers and the *Nocardia* probe. Fluorescent signal measured at 618–660 nm corresponding to the Cy5 fluorophore of the *Nocardia* probe. **F**: qPCR using HSP65 primers and the *Streptomyces* probe. Fluorescent signal measured at 465–510 nm corresponding to the FAM fluorophore of the *Streptomyces* probe. In panels **C-F**, qPCRs were performed with 5 ng of DNA derived from *M. mycetomatis* CBS109801, *F. senegalensis* CBS 197.79, *T. grisea* CBS135984, *S. boydii* CBS 620.16, *M. romeroi* CBS135987, *A. madurae* DSM43067, *A. pelletieri* DSM43383, *S. somaliensis* DSM40738, *S. sudanensis* DSM41923, *N. brasiliensis* ATCC19296 and *S. aureus* ATCC29213

A 129 bp amplicon was obtained when the DNA of the five most common causative agents of eumycetoma was amplified with the designed ITS primers by classical PCR (Fig. 1A). No amplification was noted for the bacterial causative agents. A 103 bp amplicon was obtained when the DNA of the *Actinomadura*, *Nocardia* and *Streptomyces* species were amplified with the primers designed on the *HSP65* gene with classical PCR (Fig. 1B). No amplification was obtained for *S. aureus*,* M. tuberculosis* and the fungal species. Due to the size differences between the fungal and bacterial amplicons, it was also possible to multiplex them in classical PCR and differentiate actinomycetoma from eumycetoma based on the size of the amplicon (Supplementary Fig. 1).

### The probes developed on ITS and *HSP65 *were able to detect fungal, *Actinomadura*,* Nocardia *and* Streptomyces *DNA respectively

**Fig. 2:**
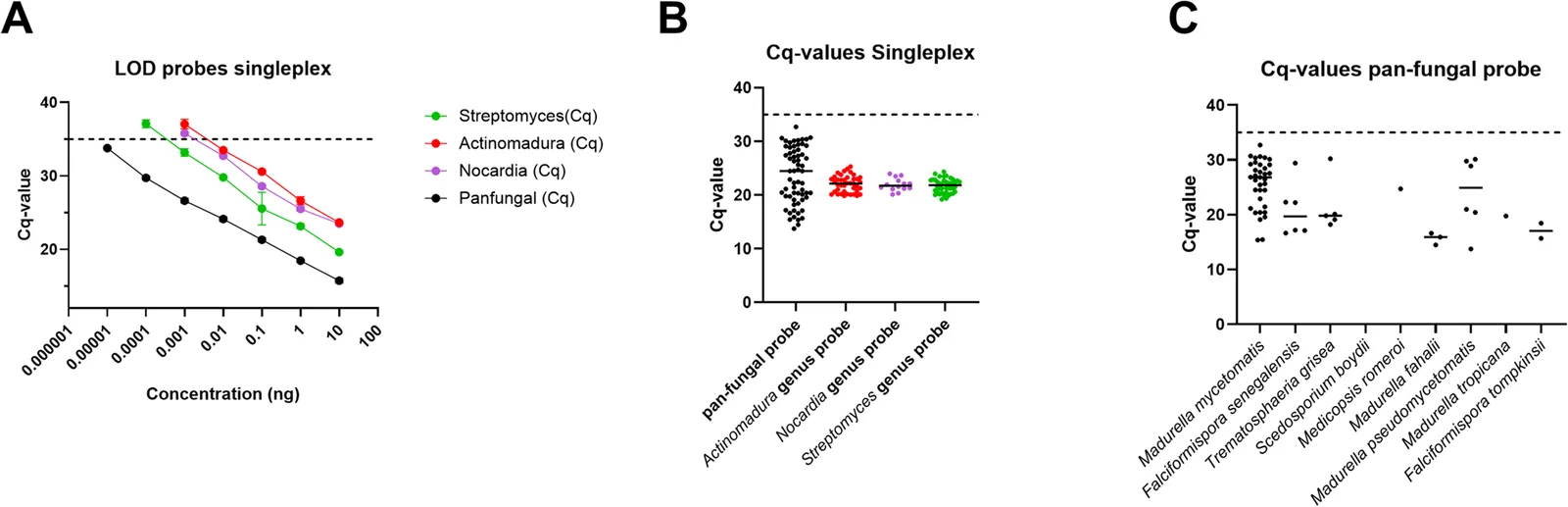
Limit of detection of the probes. **A**. Probes tested in singleplex against DNA derived from *M. mycetomatis* CBS109801, *(A) madurae* DSM43067, *N. brasiliensis* ATCC19296 and *S. somaliensis* DSM40738. **B**. Cq-values of all fungal and bacterial strains tested. **C**. Cq-values of the fungal probe per species

Next, the fungal and bacterial probes were evaluated by qPCR. The number of cycles was set at 35; the time needed to complete these cycles was less than 1 h. As shown in Fig. 1C, only the fungal species had a positive signal with the fungal probe. As shown in Fig. 1D, E and F, the *Actinomadura*,* Nocardia* and *Streptomyces* probes were also genus-specific in this small subset of strains. Next, we tested the entire strain collection (Supplementary Table 1). The ITS probe gave a positive signal for all 63 fungal DNA samples, while none of the bacterial DNA samples showed amplification, resulting in a sensitivity and specificity of 100%. The mean Cq-value for the pan-fungal probe was 23.57 (range 13.74–32.73) when 5 ng of DNA was used. The *Actinomadura* probe gave a positive signal for all 41 *Actinomadura* DNA samples, the *Nocardia* probe gave a positive signal for all 14 *Nocardia* samples and the *Streptomyces* probe gave a positive signal for all 34 *Streptomyces* samples. The mean Cq-values for the *Actinomadura* and *Nocardia* and *Streptomyces* probes were 22.08 (range 19.85–25.33), 21.94 (range 20.11–23.99) and 21.72 (range 19.23–24.34), respectively (Fig. 2B; Table 2 and Supplementary Table 1). No cross-reactivity was noted with the fungal strains, however some cross reactivity was noted with the *Actinomadura* and *Streptomyces* probe. The *Actinomadura* probe gave a positive signal for all 41 *Actinomadura* samples, but also for the *Sphaerosporangium* sp (Cq-value of 20.6). This resulted in a sensitivity of 100% and a specificity of 99.12%. The *Streptomyces* probe gave a positive signal for all 34 *Streptomyces* samples, but also for 5 *Actinomadura* species and the *Actinocorallia weidayensis* isolate. A positive qPCR was noted with *A. weidayensis* (*n* = 1), *A. cremea* (*n* = 1), *A. geliboluensis* (*n* = 1), *A. hallensis* (*n* = 1) and *A. welshii* (*n* = 2) (Supplementary Table 1). Of these species, only *A. welshii* was a reported mycetoma causative agent (Table 1) [23]. The mean Cq-value for the *Streptomyces* probes was 21.72 (range 19.23–24.34). The five *Actinomadura* species had a mean Cq-value of 22.67 (range 22.11–23.60). Due to this cross-reactivity, a sensitivity of 100% was noted and a specificity of 95.04%.

### The limits of detection of the qPCRs

Next, the limit of detection for each of the probes was determined. As shown in Fig. 2, in singleplex, 0.000002 ng of DNA was reliably detected using the pan-fungal probe. For the actinomycetoma probes, the LOD was 0.004 ng bacterial DNA for the *Actinomadura* genus probe, 0.002 ng for the *Nocardia* genus probe and 0.0004 ng for the *Streptomyces* genus probe (Fig. 2A; Table 3 and Supplementary Table 1). Furthermore, it was noted that especially for the pan-fungal probe a large range of Cq-values was obtained. Therefore, the Cq-values were also plotted against the fungal species. As can be seen in Fig. 2C on average higher Cq-values were noted for the *Madurella* species and *Medicopsis romeroi*.

**Table 3:** Limit of Detection (LOD), mean Cq-value and sensitivity and specificity for the singleplex and multiplex assays

	LOD (ng)	Mean Cq-value (range)	Sensitivity (%)	Specificity (%)
**P** **an-fungal probe**				
Singleplex	0.000002	23.57 (13.74–32.73)	100	100
Quadruplex	0.007	28.99 (16.43-37.00)	95.24	100
African triplex	0.0004	25.63 (15.10–34.10)	100	100
Latin-American triplex	0.002	26.65 (16.40–34.10)	100	100
Actinomycetoma triplex	NA	NA	NA	NA
***Actinomadura*** ** probe**				
Singleplex	0.004	22.08 (19.85–25.33)	100	99.12
Quadruplex	0.007	23.26 (20.74–26.34)	100	99.12
African triplex	0.0007	22.77 (20.54–25.76)	100	99.12
Latin-American triplex	0.01	24.87 (23.12–27.87)	100	99.12
Actinomycetoma triplex	0.005	24.25 (21.56–26.98)	100	99.12
***Nocardia*** ** probe**				
Singleplex	0.002	21.94 (20.11–23.99)	100	100
Quadruplex	0.006	23.44 (21.98–24.45)	100	100
African triplex	NA	NA	NA	NA
Latin America triplex	0.02	25.77 (24.65–27.76)	100	100
Actinomycetoma triplex	0.002	23.45 (21.11–28.90)	100	100
***Streptomyces*** ** probe**				
Singleplex	0.0004	21.72 (19.23–24.34)	95.04	100
Quadplex	0.04	22.48 (21.98–24.45)	96.04	100
African triplex	0.01	22.21 (19.78–24.75)	96.04	100
Latin America triplex	NA	NA	NA	NA
Actinomycetoma triplex	0.002	23.61 (20.11–25.11)	96.3	100

Table 2: Limit of Detection (LOD), mean Cq-value and sensitivity and specificity of the singleplex and multiplex assays

### Multiplexing the probes

Instead of running four singleplex qPCRs, it is more efficient to run these qPCRs in multiplex. In the design, the dyes of the probes were selected in such a way that a quadruplex could be applied. As can be seen in Table 3, the limit of detection of the fungal probe increased 3500 times in the quadruplex compared to the singleplex. Furthermore, the DNA of 3 *M. mycetomatis* isolates could no longer be detected in the quadruplex. This resulted in a sensitivity of 95.24% and a specificity of 100.0%. For the *Actinomadura* genus, *Nocardia* genus and *Streptomyces* genus probes the reduction in LOD was less (Table 3) and all of the samples could still be detected with qPCR, resulting in a sensitivity and specificity of 100%.

To improve the performance of the multiplex assay, we narrowed down the quadruplexes to triplexes. In total, three triplexes were designed: one that included the causative agents most prevalent in Africa, one that included the causative agents most prevalent in Latin-America and one that only included the bacterial causative agents. As shown in Table 3, in all triplexes all DNA samples were again detected by their corresponding probes, resulting in a 100% sensitivity and specificity. The LOD for the pan-fungal probe remained lower than in the singleplex, but no fungal samples were missed. The LOD for most of the bacterial samples remained in the same range as in the singleplex. Despite the differences in the LOD and the Cq-values when the quadruplex and triplexes were compared to the singleplex, the diagnostic sensitivity and specificity, remained the same (Table 3).

### Validating the qPCR on mycetoma grains

To validate the African triplex on clinical material, the qPCR was performed on DNA isolated from grains obtained from 10 different patients (Table 4). As can be seen in this table, only for one patient infected with *A. madurae* a double signal was obtained. For all the other samples the qPCR identification was correct, resulting in a sensitivity of 100% and a specificity of 83.3% for the fungal probe and a sensitivity and specificity of 100% for the *Actinomadura* probe using clinical samples.

**Table 4:** Validation of the African multiplex with 10 grain samples

Species	Grain	Cq-value fungal probe	Cq-value Actinomadura genus probe	Cq-value Actinomadura genus probe	Cq-value Actinomadura genus probe
*Actinomadura madurae*	DK57	16.78	22.6	> 35	> 35
*Actinomadura madurae*	121	> 35	24.38	> 35	> 35
*Actinomadura pelletieri*	DK7	> 35	27	> 35	> 35
*Actinomadura pelletieri*	DK8	> 35	30.17	> 35	> 35
*Actinomadura pelletieri*	DK26	> 35	32.98	> 35	> 35
*Madurella mycetomatis*	107	34.23	> 35	> 35	> 35
*Madurella mycetomatis*	115	29.32	> 35	> 35	> 35
*Madurella mycetomatis*	DK45	25.4	> 35	> 35	> 35
*Madurella pseudomycetomatis*	DK59	17.81	> 35	> 35	> 35
*Madurella pseudomycetomatis*	DK63	32.56	> 35	> 35	> 35
Sensitivity		**100%**			
Specificity		**83.3%**			

## Discussion

**Fig. 3:**
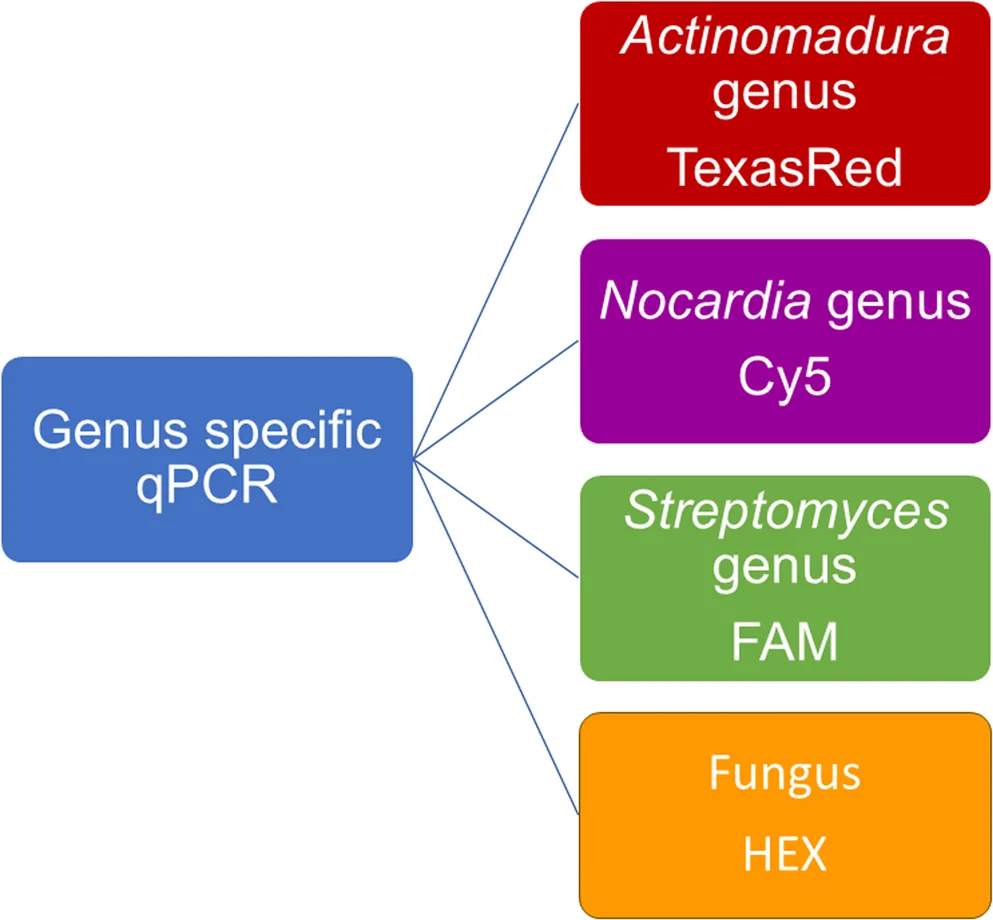
qPCR scheme to differentiate eumycetoma from actinomycetoma

The multiplex described here (Fig. 3) adheres to the minimal requirements set in the WHO TPP for a mycetoma diagnostic assay as it can differentiate actinomycetoma from eumycetoma within a day, and can be performed in a peripheral health facility or national diagnostic testing laboratory [7]. Additionally, this qPCR also allows identification to the genus level for the Actinomycetoma causative agents. In the current design, the probes appeared quite specific, but some cross-reactivity was noted with the *Actinomadura* and *Streptomyces* genus probe with cultured isolates and with fungal probe in grain samples. The *Actinomadura* probe was also positive for the *Sphaerosporangium* sp. and the *Streptomyces* probe was also positive for *A. cremea*,* A. hallensis*,* A. welshii* and *A. weidayensis.* Of these only *A. welshii* was described as a causative agent of actinomycetoma [23]. At the time of the design of this qPCR, this was not known and no sequences for *A. welshii* were available.

There was also a difference in Cq-values for the bacterial and fungal species. On average, the Cq-values for the actinomycetes were much lower than for the fungi. This difference is most likely due to the difference in genome size. The genomes of *S. somaliensis*,* S. sudanensis*,* A. madurae* and *A. pelletieri* range from 5.01 Mbp to 10.3 Mbp [24], while the genomes of *M. mycetomatis* and *M. fahalii* range from 35.7 Mb to 40 Mb [25–28]. This means that, on average, 4–7 times more copies of a single copy gene are present in 5 ng bacterial DNA compared to 5 ng fungal DNA. Furthermore, the ITS gene is a multi-copy region for which the copy number varies per species. *Madurella* species belong to the fungal order of the Sordariales, and fungi belonging to this order have, on average, 2.9 copies of the ITS region in their genome [29]. *M. mycetomatis* itself has only 1 [29]. *Falciformispora species*,* T. grisea* and *M. romeroi* belong to the fungal order of the Pleosporales which have on average 17.1 copies of the ITS region in their genome [29]. This difference in copy number also explains the large variation we noted in Cq-value with our pan-fungal probe.

The quadruplex qPCR designed with these primers and probes did result in a significant increase in Cq-values for the ITS probe only. This increase was not noted in the three triplexes designed. The increase could be the result of either oligonucleotide cross-reactivity, due to a high degree of similarity between one or more oligos, or by cross-talk between overlapping emission wavelengths, as was previously noted for other quadruplexes [30]. It could also be machine-dependent. For our settings, the problem was solved when the quadruplex was transformed into three different triplexes, each with its own specific use. Due to the variation in etiology worldwide, the quadruplex is not needed in most endemic regions [2]. In Africa, *Nocardia* species are not encountered and the triplex differentiating eumycetoma from actinomycetoma caused by *Streptomyces* and *Actinomadura* species would be able to detect > 95% of all causative agents [2]. In Latin-America, *Streptomyces* species are not encountered and therefore the triplex differentiating eumycetoma from actinomycetoma caused by *Actinomadura* and *Nocardia* will be enough. For most non-endemic countries, one could choose to run either a fungal singleplex and the bacterial triplex together or one of the region-based triplexes to identify the causative agent to the genus level. When the multiplex qPCR was validated against 10 grain samples, we were able to demonstrate the *Actinomadura* genus probe was able to identify all 5 *Actinomadura* grains. Only in a single grain *Actinomadura* grain sample a false positive fungal qPCR signal was obtained, resulting in a 100% sensitivity and 83.3% specificity for the fungal probe. Since dual mycetoma infections are rare, the *Actinomadura* probe is directed towards a genus, and the fungal probe is universal, the fungal probe most-likely detected a fungal superinfection. Indeed, due to the large taxonomic diversity of the fungal agents able to cause mycetoma, the pan-fungal probe was designed to detect all these fungal causative agents. The downside is that it will also detect dermatophytes which could be present on the skin. Therefore, in this specific case a dermatophyte or a fungal contaminant could be the cause of the signal. In cases where such dual signals are obtained, a second (q)PCR or sequencing of the ITS and 16 S barcoding genes can be used to identify the causative agent. For *M. mycetomatis* [13, 14], *M. pseudomycetomatis* [13], *M. tropicana* [13], *M. fahalii* [13], *F. senegalensis* [14], and *F. tompkinsii* [14] qPCRs are available. For actinomycetoma causative agents these qPCRs are currently being developed. Furthermore, beyond mycetoma, several other infectious and non-infectious conditions, such as cutaneous tuberculosis [31] and botryomycosis [32] can present with clinical features similar to mycetoma, posing diagnostic challenges. These can be effectively ruled out using this molecular platform and thereby preventing misdiagnosis and inappropriate therapy.

Since we were able to validate the performance of the qPCR only on 10 grain samples in Senegal, and fungal cross-contamination could occur, it is important to validate this qPCR on more patient samples and preferably in other endemic regions too with different etiologies too. In the current cohort, only fungal or *Actinomadura* grains could be validated. Therefore additional validation, especially in regions where *Nocardia* spp and *Streptomyces* spp are more common, is needed.

In conclusion, we developed a multiplex qPCR assay that can differentiate actinomycetoma from eumycetoma (Fig. 3). This is a significant advancement in the global effort to control and monitor mycetoma. Rapid and accurate differentiation between actinomycetoma and eumycetoma enables more precise mapping of disease distribution and prevalence, thereby informing targeted public health interventions. Moreover, the ability to identify causative agents swiftly allows for better understanding of regional variations in etiological profiles, facilitates timely treatment initiation, and helps reduce disease burden—particularly in resource-limited settings where mycetoma is endemic. As the assay becomes integrated into surveillance programs, it will also enhance data collection on pathogen distribution and resistance patterns, ultimately strengthening epidemiological research and guiding policy decisions aimed at improving disease management and progression toward eventual control or elimination of this neglected disease.

## Supplementary Information

Below is the link to the electronic supplementary material.

Supplementary Material 1 (XLSX 61.8 KB)

Supplementary figure 1 (PNG 223 KB)

Supplementary Material 2 (TIF 1.31 MB)

## Data Availability

All data is available in the manuscript or its supplementary tables.
